# Development of guidelines to reduce, handle and report missing data in palliative care trials: A multi-stakeholder modified nominal group technique

**DOI:** 10.1177/02692163211065597

**Published:** 2022-01-17

**Authors:** Jamilla A Hussain, Ian R White, Miriam J Johnson, Anthony Byrne, Nancy J Preston, Andy Haines, Kathy Seddon, Tim J Peters

**Affiliations:** 1 Wolfson Palliative Care Research Centre, University of Hull, Hull, UK; 2 MRC Clinical Trials Unit, University College London, London, UK; 3 Marie Curie Palliative Care Research Centre, School of Medicine, Cardiff University, Cardiff, UK; 4 International Observatory on End of Life Care, Lancaster University, Lancaster, UK; 5 Department of Public Health, Environments and Society and Department of Population Health, London School of Hygiene and Tropical Medicine, London, UK; 6 Marie Curie Research Voice, Wales Cancer Research Centre, Cardiff University, Cardiff, UK; 7 Population Health Sciences, Bristol Medical School, University of Bristol, Bristol, UK

**Keywords:** Missing data, lost to follow up, randomised controlled trials, palliative care, palliative medicine, guideline

## Abstract

**Background:**

Missing data can introduce bias and reduce the power, precision and generalisability of study findings. Guidelines on how to address missing data are limited in scope and detail, and poorly implemented.

**Aim:**

To develop guidelines on how best to (i) reduce, (ii) handle and (iii) report missing data in palliative care clinical trials.

**Design:**

Modified nominal group technique.

**Setting/participants:**

Patient and public research partners, palliative care clinicians, trialists, methodologists and statisticians attended a 1-day workshop, following which a multi-stakeholder development group drafted the guidelines.

**Results:**

Seven main recommendations for reducing missing data, nine for handling missing data and twelve for reporting missing data were developed. The top five recommendations were: (i) train all research staff on missing data, (ii) prepare for missing data at the trial design stage, (iii) address missing data in the statistical analysis plan, (iv) collect the reasons for missing data and (v) report descriptive statistics comparing the baseline characteristics of those with missing and observed data. Reducing missing data, preparing for missing data and understanding the reasons for missing data were greater priorities for stakeholders than how to deal with missing data once they had occurred.

**Conclusion:**

Comprehensive guidelines on how to address missing data were developed by stakeholders involved in palliative care trials. Implementation of the guidelines will require endorsement of research funders and research journals.

**What is already known about the topic?**

- Missing data are a significant problem in palliative care trials, with nearly a quarter of primary outcome data estimated to be missing at the primary follow-up point, and evidence that this may introduce bias.
- Guidance on how to reduce and manage missing data in clinical trials has mostly focussed on statistical methods to handle missing data.

**What this paper adds**

- Multiple stakeholders, including patient and public research partners and clinicians, developed detailed guidelines on how to (i) reduce, (ii) handle and (iii) report missing data in palliative care clinical trials.
- Recommendations on how to reduce missing data, including preparing for missing data, were considered to be more important than those on how to deal with missing data once they had occurred.
- Understanding the reasons for missing data was an important cross-cutting theme prioritised by stakeholders.

**Implications for practice, theory or policy**

- Stakeholder involvement aimed to increase the acceptability and feasibility of the guidelines to end-users.
- Asking participants or proxies about the reasons for missing data was considered to be acceptable and important.
- Implementation of the guidelines will require endorsement by research funders and journal editors.

## Introduction

Missing data can introduce bias, reduce the power of a study to detect a difference between treatment arms if one exists and reduce the generalisability of study findings.^1,2^ Addressing missing data is therefore essential to reduce waste in research^3^ and improve its value to clinical practice.

Missing data are a particular problem in palliative care trials. A systematic review of 108 palliative care trials estimated that 23% (95% CI 19%–27%) of primary endpoint data were missing.^4^ This compares with only 6%–10% of primary outcome missing data in clinical trials published in major medical journals.^5–7^ In terms of statistical power, in trials that provided sufficient information, 62% of palliative care trials did not achieve the pre-specified minimum sample size once missing data were taken into account.^8^ Moreover, the amount and reasons for missing data differed between trial arms, suggesting that missing data may have biassed the study findings.^4^ Despite this, only 3% of palliative care trials reported the assumed mechanism of missing data, and 16% reported a missing data sensitivity analysis. The total amount of missing data and discussion of the impact were also incompletely reported.^9^

Guidelines on how to reduce and manage missing data in clinical trials to date have been limited. The National Research Council,^2^ the International Conference on Harmonisation of Technical Requirements for Registration of Pharmaceuticals for Human Use,^10^ and the European Medicines Agency^11^ have produced guidelines, but these focus predominantly on statistical methods to handle missing data and address statisticians and methodologists.

In 2010, the Methods Of Researching End of Life Care (MORECare) collaboration identified missing data as a particular issue in palliative care research and provided broad recommendations based on expert opinion.^12^ However, these guidelines were not comprehensive and lacked detail, especially in terms of how to implement the recommendations,^12^ and some stakeholders were omitted from the process. To address missing data effectively, all involved in the design, conduct and reporting of a clinical trial – including the research participants where possible and their clinicians – must understand why missing data matter and their role in addressing this issue. Therefore, any guidelines must recognise and encompass the views, concerns and ideas of all members of the multidisciplinary team involved in the successful completion of a clinical trial and be accessible to all.

We therefore used a modified nominal group technique with key stakeholders involved in palliative care clinical trials to develop guidelines on how to address missing data. Three guidelines were developed which covered, namely, how to reduce, handle and report missing data in palliative care clinical trials.

This paper reports the methods used to develop the guidelines and presents a summary of the recommendations (details will be available at https://www.mariecurie.org.uk/ by searching “missing data guidelines”).

## Methods

### Setting

A 1-day workshop was commissioned by Marie Curie to take place before the UK Marie Curie conference in 2017. Formal ethical review was not undertaken for this expert consensus guideline development process, and attendance at the workshop was taken as implied consent.

### Design

A modified nominal group technique was used to develop the recommendations which allowed delegates to develop ideas, identify priorities and inform the guidelines in a structured manner.^13^ Five steps were followed: (i) summary of the evidence, (ii) silent generation of ideas, (iii) contributing and developing ideas by structured groups, (iv) voting,^13^ (v) writing the guidelines. The steps and rationale for the design are specified in Table 1.

**Table 1.** Nominal group technique process and rationale.

Method	Process	Rationale
Summary of the evidence	Draft recommendations for consideration were developed based on the current evidence. • These covered how to reduce, handle and report missing data in palliative care trials. • Based on a literature review, systematic review of 108 palliative care trials,^4^ individual participant level data analysis of 10 phase 3 palliative care trials and interviews with research personnel involved in palliative care trials (unpublished).	To provide a framework to stimulate discussion and debate about what should be included in the guidelines. This was thought to be necessary as this was not a topic area all participants were familiar with and delegates differed in their areas of expertise.
	Delegates were sent a pre-workshop pack detailing the aims, key questions, draft recommendations and format of the workshop. As well as a glossary of technical terms and a lay-summary of the issues missing data pose to palliative care trials for those less familiar with the topic area.	To allow reflection prior to the workshop, thus enabling individual generation of ideas prior to the meeting.
	At the start of the workshop there were three presentations which covered (i) evidence of why missing data matter in palliative care research, (ii) challenges of trying to reduce missing data in palliative care trials and (iii) an overview of the methods to handle missing data at the statistical analysis stage. The presentations were designed to ensure all members of the workshop could understand the content.	To ensure all delegates had a shared understanding of the three areas under consideration.
	Draft recommendations were presented to the group. The delegates were reminded of the purpose the meeting and why each stakeholder was important to tackling missing data in trials. The delegates were advised that as key stakeholders in the design and conduct of palliative care trials, the guideline group wanted them to develop the guidance using the drafted recommendations only as a potential framework which they were to critique, amend and if necessary transform to develop a guideline they would find useful and usable.	To ensure the guidelines reflected the ideas, framing and preferences of the different stakeholders.
Silent generation of ideas	The delegates were informed at the start of the day that the aim was for them to generate ideas. They were advised to document thoughts/comments throughout the morning sessions and then prior to the afternoon session to take 10–15 min to reflect on each of the guidelines that had been presented to generate their own thoughts, queries and ideas.	Enable all participants to think about their own ideas, reflections prior to sharing and working on the guideline as a group. This helped to generate original ideas and provided time for delegates to clarify their own thoughts.
Contributing and developing ideas	In the afternoon, the delegates were split into 5 groups of 8–10 delegates. The groups were mixed, however groups 1 and 2 were predominantly representative of patient and public research partners and clinicians, groups 3 and 4 statisticians and methodologists, and group 5 palliative care researchers and trialists. Each group was given two guidelines to review with groups 1 and 2 addressing how to reduce missing data first, groups 3 and 4 how to handle missing data, and group 5 how to report missing data.	The groups were split to ensure the stakeholders considered the most relevant guidance, to which they could contribute the most, in detail. It also helped to ensure members of the groups felt comfortable to share their ideas and protected against the discussion being dominated by certain individuals. However, it was stressed throughout the process that everyone’s views mattered for each of the guidelines.
	Each group had a facilitator who was provided with a briefing which included instructions to ensure all recommendations were understood and discussed, and that all members of the group were given an opportunity to contribute equally. A round robin was suggested where each delegate introduces themselves and shares their views on any recommendations they (i) disagreed with, (ii) would amend, (iii) would add and/or (iv) they feel strongly should be included. Each facilitator had sufficient knowledge of the topic under consideration and experience of group moderation.	To ensure the facilitator was aware of the aims, process and how to best facilitate the group such that the outcomes reflected the views of the group.
	Each draft recommendation was printed on an A4 card and the delegates were encouraged to amend and add comments to the cards with new recommendations added to separate cards. Recommendations could also be removed.	To enable the groups to visualise the draft recommendations, physically rearrange them and amend them directly. This enabled the participants to clarify and express their understanding and opinions of the recommendations/ideas, and to explore the reasoning underlying their perspective.
	Each group had 90 min to discuss the recommendations. Scribes were present in each group to capture the discussion.	Typically, groups used 45 min per topic. Each group had the option to discuss two topics therefore the afternoon session was extended to 90 min. This aimed to give groups enough time to develop their ideas and if they wanted they had the option to work on one guideline for the entire time. This enabled the richness and depth of discussion to develop and the discussion from one guideline could be used to inform the development of the second guideline. Scribes captured the basis of the decisions.
	Each group was asked to decide on their final list of recommendations.	To focus the discussion, keep the group on task and to ensure the views of the group would directly influence the development of the guidance.
Voting	Cards with the amended or new recommendations were placed on the walls ensuring visibility to all delegates.	Enabled all delegates to visualise the recommendations as a whole.
	All delegates and facilitators were invited to put a colour coded sticker next to the top three recommendations they considered should be included, as a matter of priority, in the guidance they discussed. They also had the opportunity to add stickers to the other guidelines even if these had not been discussed specifically in their group.	To identify which recommendations delegates considered to be the most important.
	Each group presented their discussion and recommendations to the rest of the group, whilst considering the visible cues from the voting.	Everyone could consider each guideline, the detailed discussion within the group, whilst taking into account how important the entire group considered each recommendation to be.

Nominal group techniques seeks to generate a range of ideas and so key informants should be selected to participate.^13^ Participants were therefore purposively sampled based on their expertise and included patient and public involvement (PPI) research partners, palliative care clinicians, palliative care trialists and methodologists including statisticians (Table 1). The steering group identified potential participants who were contacted via email by the workshop organisers. Potential participants were also asked to recommend any other potential participants. The PPI research partners had a range of expertise, including experiencing advanced chronic illness, being a carer for someone with a life-limiting illness and being involved as PPI partners and/or participants in palliative care trials. Although in the majority the palliative care trialists also had worked, or currently worked, as palliative care clinicians, clinicians without an academic trials background were specifically recruited to provide the perspective of clinicians supporting patients through studies. Methodologists with expertise in missing data analyses and trial design and conduct both within and outside of palliative care were sampled. This included methodological leaders in this area. A priori a cap of 60 delegates in total was set to ensure the groups could engage in detailed discussion from all participants^13^ – if the cap was reached the steering group would decide on which delegates to include to maximise the diversity of expertise available.

### Data analysis and synthesis

Raw data from voting were entered into an Excel spreadsheet to provide the frequency of votes for each recommendation by delegate role (Table 2).

**Table 2.** Original votes for the recommendations.

Guideline	Recommendation	PPI/clinicians	Palliative care researchers	Methodologists/Statisticians	Total (max 51^a^)	Rank
Reducing missing data	Prepare for missing data at the trial design stage	11	5	7	23	2
	Train all research staff	15	4	8	27	1
	Discuss missing data with participants before they consent to start the trial		1	1	2	
	Collect the reasons for missing data	6	6	5	17	4
	Monitor missing data during the trial	3	2	4	9	
	Resource the trial adequately to reduce missing data		1		1	
Handling missing data	Address missing data in the statistical analysis plan	11	4	6	21	3
	Prepare for missing data analysis at the trial design stage	6	1	7	14	
	Inflate the sample size	2	2	9	13	
	Collect the reasons for missing data to inform the assumptions about the missing data mechanism	3	1	11	15	
	Consider whether any auxiliary variables should be collected			1	1	
	Consider how to handle truncated data due to death				0	
	Explore the missing data	2	1	10	13	
	Decide which assumptions about the missing data mechanism are plausible				0	
	Choose and conduct primary analyses valid under the missing data assumption	1	1	4	6	
	Conduct missing data sensitivity analyses	2	2	10	14	
Reporting missing data	Report the missing data analytical approach				0	
	Report the justification of the missing data analytical approach	6	7	1	14	
	Report details of the statistical methods used to handle missing data				0	
	Report how truncated data due to death were handled with a justification for the method(s)				0	
	Report the amount of missing data in those alive and those who died		3	3	6	
	Report detailed reasons for missing data in each trial arm	7	4	5	16	
	Report descriptive statistics comparing the baseline characteristics of those with missing data and observed data	7	3	7	17	4
	Report the findings of the investigations of the missingness mechanism				0	
	Report results of the missing data sensitivity analyses		3	7	10	
	Discuss the impact of missing data	6	5	5	16	

PPI: patient and public involvement research partner.

a Each attendee could vote for their top three recommendations – each recommendation therefore had the potential for 51 votes.

Notes from the scribes were transcribed and coded based on the principles of thematic analysis^14^ by one researcher (JH) who developed the initial framework using NVivo (Supplemental Material 1). The transcripts and coding framework were reviewed and clarified with a second researcher (MJJ) from which reducing, handling and reporting missing data themes were identified and used to generate a draft outline for the guidelines. Coded recommendations were rearranged by theme and duplicates were combined. The original language used by participants was used where possible, with amendments for clarity. The group facilitators reviewed the frequency of votes for each recommendation, the generated themes and draft guidelines which highlighted areas of uncertainty and contradictions. The facilitators provided suggestions and comments, following which the draft guidelines were updated. The guidelines were reviewed by a development group comprising a patient research partner, clinicians, palliative care trialists and methodologists including statisticians; four iterations were drafted before the guidelines were approved by all members.

The original workshop notes, codes and votes were then reviewed by two researchers to ensure the recommendations reflected the ideas, perspectives and priorities of the participants. As the themes for the recommendations had evolved, where some were amalgamated or amended, the original votes, informed by the transcribed discussions, were re-matched to the current recommendations to ensure the recommendations included and prioritised the delegates’ original priorities.

## Results

Table 3 summarises how the recommendations were developed. Seventy-five participants were contacted and 39 attended the workshop (65% female, all but one based in the UK) in addition to eight steering group members and four organisers and scribes.

**Table 3.** Guideline development.

Group	Recommendation considered	Number of recommendations following review of evidence	Number of main and sub-recommendations following Nominal Group Technique	Suggested changes to recommendations through Nominal Group Technique		Number of recommendations developed by guideline development group
1	Reducing missing data	10	Main 5 Sub 24	New	2	Main 7 Sub 24
				Amalgamated/split	7	
				Amended	4	
				Unchanged	0	
2	Reducing missing data	10	Main 12 Sub 32	New	2	
				Amalgamated/split	0	
				Amended	7	
				Unchanged	2	
3	Handling missing data	13	Main 12 Sub 20	New	0	Main 9 Sub 13
				Amalgamated/split	1	
				Amended	11	
				Unchanged	0	
4	Handling missing data	13	Main 12 Sub 16	New	0	
				Amalgamated/split	0	
				Amended	10	
				Unchanged	2	
5	Reporting missing data	7	Main 6 Sub 13	New	0	Main 12 Sub 8
				Amalgamated/split	1	
				Amended	5	
				Unchanged	1	

The qualitative analysis of the scribes’ notes generated 54 codes, from which 27 themes were identified (6 reducing, 10 handling and 11 reporting missing data; Supplemental Material 1). Based on the recommendations across groups, analysis of scribe notes, voting and expertise, the guideline development group developed 7 main recommendations for reducing missing data (with 24 sub-recommendations), 9 for handling missing data (13 sub-recommendations), and 12 for reporting missing data (8 sub-recommendations).

The top five recommendations scored by the participants at the workshop are shown in Table 2. The recommendations are summarised in Tables 4 to 6 with their re-matched scoring based on the original votes.

**Table 4.** Recommendations for reducing missing data.

No.	Recommendation	Score^a^
1	*Prepare and plan for how to reduce missing data at the trial design and protocol development stage.*	23
2	*Resource the trial adequately to reduce missing data.*	1
3	*Train all research staff to understand the risks to the integrity of the trial posed by missing data and how to reduce missing data.*	27
4	*Discuss the value of complete data and how to reduce missing data with participants before they consent to enter the trial.*	2
5	*Collect the reasons for missing data.*	17
6	*Distinguish participants who want to withdraw from providing any further data from participants who wish to withdraw from part of the study protocol but consent to ongoing data collection or access.*	0
7	*Monitor and address missing data during the trial.*	9

a Re-matched scores based on original votes, max *n* = 51.

**Table 5.** Recommendations for handling missing data.

No.	Recommendation	Score^a^
1	*Include a statistician in the trial team during the design, conduct and analysis stages of the study.*	21
2	*Decide how missing data will be handled in the design and conduct of the study and in its analysis, and report these decisions in the protocol and statistical analysis plan.*	21
3	*Prepare for missing data analysis at the trial design stage.* This includes collecting the reasons for missing data and considering whether any auxiliary variables (i.e. variables not in the main statistical model, but which are associated with missing data) should be collected.	30
4	*Inflate the sample size to account for expected missing data in order to achieve the number of participants necessary to power the study adequately.*	13
5	*Consider how to handle data truncated due to death.*	0
6	*Explore the nature of the missing data in order to inform the missing data analyses.*	13
7	*Decide which assumptions about the missing data mechanism are plausible for primary and secondary outcome* analyses *in light of Recommendation 6.*	0
8	*Choose and conduct primary* analyses *that provide valid inferences under the missing data assumptions chosen in Recommendation 7, taking into account any auxiliary variables in the model.*	6
9	*Conduct missing data sensitivity* analyses *that assess the sensitivity of the results to plausible departures from the primary missing data assumption. These should include an exploration of missing not at random (MNAR) assumptions if plausible.*	14

a Re-matched scores based on original votes, max *n* = 51.

**Table 6.** Recommendations for reporting missing data.

No.	Recommendation	Score^a^
Methods		
1	*Report strategies used to reduce missing data throughout the trial process.*	0
2	*Report if and/or how the original sample size calculation accounted for missing data and the justification for these decisions. Report if and/or how the sample size was reassessed during the course of the trial.*	0
3	*Report the assumption about the missing data mechanism for the primary analysis and the justification for this choice, for all outcomes.*	14
4	*Report the method used to handle missing data for the primary analysis and the justification for the methods chosen, for all outcomes. Include whether or which auxiliary variables were collected and used.*	0
5	*Report the assumptions about the missing data mechanism and methods used to conduct the missing data sensitivity* analyses *for all outcomes, and the justification for the assumptions and methods chosen.*	14
6	*Report how data that were truncated due to death were handled with a justification for the method(s) (if relevant).*	0
Results		
7	*Report the numbers and proportions of missing data in each trial arm.*	6
8	*Report the reasons for missing data in each trial arm.*	16
9	*Report a comparison of the characteristics of those with observed and missing data.*	17
10	*Report the primary analysis based on the primary assumption about the missing data mechanism, for all outcomes.*	0
11	*Report results of the missing data sensitivity* analyses *for all outcomes. As a minimum a summary of the missing data sensitivity* analyses *should be reported in the main paper with the full results in the supplementary material.*	10
Discussion		
12	*Discuss the impact of missing data on the interpretation of findings, considering both internal and external validity.*	16

a Re-matched scores based on original votes, max *n* = 51.

### Reducing missing data recommendations

Recommendations for reducing missing data are given in Table 4.

*1.* *Prepare and plan for how to reduce missing data at the trial design and protocol development stage.*

This was a key priority across stakeholders. Ideas of how to reduce missing data included developing a flexible study design that facilitates data collection as the physical, psychological and/or social circumstances of the participant change. For examples, trialists should consider more than one mode of data collection such as face-to-face, telephone and electronic data collection. The need to consult members of the multidisciplinary team involved in conducting a trial on how to reduce missing data was considered to be important, in particular experienced data collectors such as research nurses. Reducing the trial burden through minimising the amount of data collected and duration of the study was suggested in keeping with the evidence base. Strategies to reduce missing data should also be evaluated to determine which are most effective.

*2.* *Resource the trial adequately to support patients, carers, clinical team members and data collectors to provide complete data.*

The attendees considered the need for additional funds to collect data across settings, as participants may move between settings such as home/care home, hospital and hospice. Also funding for the use of different modalities of data collection, incentives for sites to provide data, and recruitment of staff with a good track record for data collection were suggested.

*3.* *Train all research staff to understand the risks to the integrity of the trial posed by missing data and how to reduce missing data.*

It was recommended that training should cover why complete data are important, how to communicate with and support participants with palliative care needs to provide data, how to enter and check data and how to document the reasons for missing data.

*4.* *Discuss the value of complete data and how to reduce missing data with participants before they consent to enter the trial.*

This includes exploring participants’ concerns about the data collection process and informing them why each outcome is being collected, the importance of complete data, why collecting the reasons for missing data is important and consent for the use of proxies and/or access to their medical records if they are unable to provide data.

*5.* *Collect the reasons for missing data.*

This was identified both in the voting and the qualitative analysis as important. The recommendation did however generate debate. Some were in favour of asking participants for the reason(s) they were unable to provide data:

‘The PPI representatives on the table discussed the importance of [participants] being able to ask “why” the data was needed, but as equally it was important for [the] researcher to be able to ask participants “why” they hadn’t provided data*’. (Group 1)*

However, it was specified that consent to be asked the reasons for missing data was important:

‘The table agreed that, provided they had asked for consent to ask why, it was important [to ask the participant why they were unable to provide data]. If participants had the CHOICE not to give reasons, the researchers should have the PERMISSION to ask. The clinicians on the table agreed that this would be in compliance with Good Clinical Practice’. *(Group 1)*

There was some disagreement in how consent should be taken; however, the majority decision was that verbal consent was sufficient.

‘Some on the table felt that specific written consent was important from an ethical and pragmatic view, while others felt that circumstantial verbal consent would suffice. . . The table came to a majority decision (although not all agreed) that the consenting process could be verbally agreed’. *(Group 1)*

The burden of collecting the reasons for missing data for both the participant and data collector and the risks associated with this were also discussed. As a minimum the reasons for missing data for the primary outcome, especially at the primary endpoint, was recommended to be collected. However, this should be considered a minimum. Furthermore, the challenges of collecting the reasons for missing data, especially if the participant has completely withdrawn from the trial or becomes too unwell, were also discussed – but the need to try to collect this information as effectively as possible was still considered to be very important.

It was strongly recommended that terms such as ‘withdrawal’, ‘lost to follow-up’ or ‘dropout’ without specifying the underlying reason were avoided as they are uninformative and ambiguous.

The full recommendation is available in Supplemental Material 2.

*6.* *Distinguish participants who want to withdraw from providing any further data from participants who wish to withdraw from part of the study protocol but consent to ongoing data collection or access.*

The importance of continuing to collect data even if the participant withdraws from the intervention was stressed by methodologists to enable intention to treat analyses to be conducted.

*7.* *Monitor and address missing data during the trial.*

Monitoring the amount and reasons for missing data for each trial arm and addressing any modifiable reasons as soon as possible was recommended to minimise the impact of missing data as the trial proceeds.

### Handling missing data recommendations

Recommendations for reducing missing data are given in Table 5.

*1.* *Include a statistician in the trial team during the design, conduct and analysis stages of the study.*

Methodologists and trialists highlighted the importance of including statisticians at the start of the trial, as they have expertise on how to optimise trial design to minimise the impact of missing data, as well as how to analyse and interpret findings.

*2.* *Decide how missing data will be handled in the design and conduct of the study and in its analysis, and report these decisions in the protocol and statistical analysis plan.*

Setting out clearly how missing data will be addressed throughout the trial was considered an important step in addressing the handling of missing data.

*3.* *Prepare for missing data analysis at the trial design stage.*

This includes collecting the reasons for missing data to inform the missing data assumptions and analyses, and considering whether any auxiliary variables (i.e. variables not in the main statistical model, but which are associated with missing data) should be collected as they can reduce bias and improve the statistical power when missing data occur.

*4.* *Inflate the sample size to account for expected missing data in order to achieve the number of participants necessary to power the study adequately.*

Statisticians specified that this should include deciding on the appropriate sample size for the study without missing data, estimating the expected missing data based on evidence and expertise, inflating the sample size accordingly and re-evaluating the strategy if missing data are substantially different to that anticipated.

*5.* *Consider how to handle data truncated due to death.*

It was discussed that missing data truncated due to death presents a different issue to missing data in those alive and therefore requires different methodological approaches. Methods to impute for missing data after death were not considered to be appropriate in the palliative care setting, as the values of the outcome if death had not occurred are not meaningful for practice. Survivor-only analyses and composite approaches were discussed as alternative methods of analysis.

*6.* *Explore the nature of the missing data to inform the missing data analyses*.
  This is to understand the potential mechanisms for the missing data and includes exploring the amount, patterns and reasons for missing data as well as the distribution of variables according to whether the participant had missing data or not.
*7.* *Decide which assumptions about the missing data mechanism are plausible for primary and secondary outcome analyses* *in light of Recommendation 6.*

Based on established methods, the methodologists suggested that findings of recommendation 6 are used to inform the missing data assumption(s) for the primary and secondary outcome analyses.

*8.* *Choose and conduct primary* analyses *that provide valid inferences under the missing data assumptions chosen in Recommendation 7, taking into account any auxiliary variables in the model.*

The plausible assumptions about the missing data should inform the methods used to handle missing data. Statistician’s noted that additional considerations when choosing between different valid approaches include how much data are missing, which variables are missing, the pattern of missingness and computational efficiency.^15^

*9.* *Conduct missing data sensitivity* analyses *that assess the sensitivity of the results to plausible departures from the primary missing data assumption. These should include an exploration of missing not at random (MNAR) assumptions if plausible.*

The assumptions about the missing data mechanism cannot be verified using the data that are observed. Therefore, everyone agreed that it was important to assess the sensitivity of the findings to different assumptions about the missing data mechanism by performing a number of different sensitivity analyses that are valid under different assumptions.^16^

### Reporting missing data recommendations

The recommendations for reporting missing data are reported in Table 6.

#### Priority recommendations

Three of the top five original recommendations across all three guidelines were recommendations for reducing missing data (Table 2), including preparing for missing data at the trial design stage and training staff. Furthermore, the principal recommendation for handling missing data was to address it in the statistical analysis plan at the start of the trial. Understanding the reasons for missing data was a key cross-cutting theme across all three guidelines.

## Discussion

Comprehensive guidelines on how to better reduce, handle and report missing data in palliative care trials were developed using a modified nominal group method involving PPI research partners, clinicians, trialists and statisticians. Specifically, these included a large focus on the prevention of missing data at the design stage. Asking about and understanding the reasons for missing data was an important cross-cutting theme prioritised by stakeholders in all three guidelines.

### What this study adds

Guidelines on how to address missing data throughout the course of a trial have been developed with the inclusion of different stakeholders to widen the scope and develop relevance, depth and clarity. The variety of perspectives generated debate and allowed assumptions by different groups to be assessed and addressed. It also helped non-methodologists to understand and contribute to a topic that directly affects them, either as research participants tasked with providing data or as clinicians supporting patients through trials and as end-users of research, but is often not made accessible. This was important as missing data has ethical as well as methodological implications.^17,18^ Furthermore, it ensured that the recommendations represented the perspectives of a range of individuals who will be paramount in implementing the guidelines, thus securing ‘buy-in’ at the development stage.^19^ This will be important to influence policy and practice.^17,19^

By inviting delegates to consider all three guidelines together, we identified the prime importance of considering and actively planning for missing data before a trial starts, rather than at the point of analysis. This is a significant finding, as many of the developments in the field of missing data over the past 50 years have been to develop more sophisticated methods to handle missing data once they have occurred.^2^ However there is still little evidence on how to plan and prepare for missing data effectively and, in particular, how to reduce missing data in the first place.^21,22^

Understanding the reasons for missing data was selected as a top three priority for all three guidelines. Knowledge of the reasons for missing data is central to understand how to reduce missing data, choose and justify the statistical analysis approach and assess the accompanying risk of bias. Despite this, reporting of the reasons for missing data is poor in palliative care trials.^9,23^ Although there was support for providing participants with the opportunity to explain why they were unable to provide data, how to do this, including how to gain consent, remained contentious amongst stakeholders. Further research is required to ensure methods to collect the reasons for missing data are validated, ethical, support participants and are useful to and useable by different stakeholders.

Despite developments in the guidelines on how to handle missing data in trials over the last decade, which have included to some degree guidance on reducing missing data in the first place^2^ and recognition of the need to understand the reasons for missing data,^16^ the implementation of these aspects of the guidelines has been limited in palliative care. This is potentially because these areas have not been prioritised as key issues to understand and address, and this paper demonstrates the importance of these areas to different stakeholders.

### Limitations and strengths

To achieve high external validity of the guidelines, we tried to choose individuals with a range of experience and expertise. However, the guidelines can only represent the consensus of the individuals included and able to attend. In particular, PPI representatives involved in palliative care research were used to represent research participants’ and patients’ views, rather than patients and carers themselves. This was partially mitigated against by purposively sampling individuals with insight into the patient and participant experience as well as experience of clinical trials. Nearly half of the people contacted to take part did not attend, which may have resulted in a biassed sample of attendees, especially as participants had to attend in person which limited participants largely to those based in the UK. Our approach however did enable a range of stakeholders with different perspectives to consider the evidence, generate and share their ideas equally and help develop timely guidelines which were representative of the views of all stakeholders.

The internal validity of the guidelines is limited by the selection of background information presented to participants at the start of the workshop – although this was deliberately wide-ranging and included published and unpublished evidence both within and outside of the field of palliative care. Facilitators were briefed prior to the workshop and on the day about their role in ensuring equal participation by each member to support balance of influence within and across groups. Group think was further mitigated against by enabling silent generation of ideas, everyone sharing their ideas at the start of the group discussion and voting.

The guideline development group was selected to be diverse in expertise and perspectives and throughout the process referred back to the discussions at the workshop to ensure they were represented in decision-making. The final guidelines however were written by a selected group of individuals and delegates were not given the opportunity to review or re-score the final recommendations. This was the chosen approach as considerable time had elapsed from the workshop to the guidelines being agreed such that there was a substantial risk that the knowledge and understanding, especially of those not steeped in trial methodology, may have become less clear in that time frame and therefore the re-scoring may not have been consistent across stakeholder groups.

## Conclusion

Reducing, handling and reporting missing data is essential to improving the value of palliative care trials and therefore improving care for patients, family and carers. Comprehensive guidelines on how to achieve this are an important step to reducing the disparity in tackling missing data in this and similar fields.^24^ It is however important to note the guidelines are not a definitive endpoint, but rather are based on the current evidence, consensus of the participants and steering group expertise. To further strengthen the guidelines it is now essential for the guideline group to: (i) get feedback on the guidelines and update them as new evidence, feedback and experience emerges – the guidelines will be published on the Marie Curie website and feedback will be encouraged from users; (ii) proactively disseminate the guidelines to Clinical Trials Units conducting palliative care studies; (iii) support implementation including advocating for research funders and journal editors to endorse the guidelines; and (iv) assess the effectiveness of the guidelines in improving missing data outcomes by reviewing progress.

## Supplemental Material

sj-pdf-1-pmj-10.1177_02692163211065597 – Supplemental material for Development of guidelines to reduce, handle and report missing data in palliative care trials: A multi-stakeholder modified nominal group technique Click here for additional data file. Supplemental material, sj-pdf-1-pmj-10.1177_02692163211065597 for Development of guidelines to reduce, handle and report missing data in palliative care trials: A multi-stakeholder modified nominal group technique by Jamilla A Hussain, Ian R White, Miriam J Johnson, Anthony Byrne, Nancy J Preston, Andy Haines, Kathy Seddon and Tim J Peters in Palliative Medicine
