# Supplementary material for: Development of guidelines to reduce, handle and report missing data in palliative care trials: A multi-stakeholder modified nominal group technique
Source: Palliat Med. 2022 Jan 17;36(1):59–70. doi: 10.1177/02692163211065597 (PMC8796167; doi:10.1177/02692163211065597)
Supplement: sj-pdf-1-pmj-10.1177_02692163211065597 – Supplemental material for Development of guidelines to reduce, handle and report missing data in palliative care trials: A multi-stakeholder modified nominal group technique [file sj-pdf-1-pmj-10.1177_02692163211065597.pdf]

Supplementary material 1: Missing data workshop coding framework

Nodes

Cross-cutting	Themes that cut across the guidelines	0	0
communication	Role of communication	1	2
Funding	Role of funding	3	3
Reasons	Importance of understanding the reasons for MD and how to address this	4	7
why_impt	Why discussing reasons for MD is important	1	1
HANDLING	Themes related to handling MD	0	0
amount_md	Amount of MD	2	2
aux_var	Role of auxiliary variables in MD analysis	2	2
death_md	Importance and how to handle data truncated due to death	2	2
mech_md	Establishing mechanism of MD	2	2
res_q	Defining the research question, outcomes, and causal estimands considering MD	3	4
SA	Missing data sensitivity analyses	2	2
sample_size	Calculating the sample size considering MD	2	3
SAP	Developing a statistical analysis plan	2	2
improving_clarity	Group discussions aimed at improving clarity of recommendations	0	0
Definitions	Group discussions about definitions	2	3
Structure	Group discussion related to guidance structure	3	8
Usability	Group discussion related to improving the usability of the guidance	1	1
Detail	Group discussion about the level of detail of the guidance	1	1
Length	Limiting the length of the guidance	1	1
Wording	Group discussion on how to change the wording of the guidance	5	9
recomm_change	How the groups recommended the guidance be changed	0	0
clear+concise	Recommendation described as clear and concise, not changed	5	7
Combined	Recommendation combined with another recommendation(s)	1	1
New	New recommendation developed	2	3
not_clear	Group identified the recommendation as being unclear	3	5
Omitted	Group suggested the recommendation be omitted	0	0
REDUCING	Group discussion about the guidance to reduce MD	0	0
MDT	Role of multidisciplinary approach including all stakeholders	1	1

monitoring_md	Discussion related to monitoring MD	2	3
Participants	Role of participants to reduce md	1	1
disc_md	Role of discussing MD with participants	1	1
research_personnel	Discussion about the role of research personnel	1	1
incentives	Role of incentives	1	2
trackrecord	Role of using those with a track record of reducing MD	1	1
Training	Role of training	2	2
trial_design	Role of trial design	2	2
wd_trial_v_prot	Differentiating participants who want to withdraw from the trial vs part of the protocol	1	1
REPORT	Discussion related to reporting MD	0	0
amount_md_report	Reporting the amount of MD	1	1
baseline_charac	Reporting comparison of baseline characteristics	1	1
discuss_report	Report the implications of MD in the discussion	1	1
justification_analysis	Justify the analysis approach	1	1
mech_ix_report	Reporting the investigation of the mechanism of MD	0	0
sa_report	Reporting the MD sensitivity analysis	1	1
type_discussion	Type of discussion developed by the group	0	0
Agreement	Reported agreement with the draft recommendation	3	11
Clarifying	Group discussed how to/need to clarify the recommendation	5	10
developing	Group developed the recommendation	4	19
Debate	Group debated the recommendation	2	5
disagreement	Group disagreed with the recommendation or disagreed amongst themselves	4	4
Priority	Group identified during their discussion that the recommendation was a priority	4	9

Supplementary material 2: Recommendation for collection the reasons for missing data

Document the reasons for missing data systematically and clearly

a) Design a form to collect the underlying reasons for missing data, in collaboration with PPI research partners, clinical team members, data collectors, data managers, statisticians and experienced trialists. Assess and amend the form as part of the feasibility and/or pilot studies with feedback from participants, proxies and the trial team, and continue to review and amend the form as necessary during the trial.
b) Include the following reasons for missing data as a minimum: death, disease progression unrelated to the intervention, adverse events or reactions and reasons related to the primary outcome. If the reason is unknown, specify why this was the case.
c) Document who provided the reason for missing data, e.g. participant, proxy, data-collector, clinician, principal investigator, unknown.
d) Do not use ambiguous terms such as ‘withdrawal’ or ‘lost to follow up’ without specifying the underlying reason. Avoid the inherently ambiguous term ‘drop-out’ in all circumstances.
